# A dynamic transport model for quantification of norovirus internalization in lettuce from irrigation water and associated health risk

**DOI:** 10.1016/j.scitotenv.2018.06.158

**Published:** 2018-12-01

**Authors:** Srikiran Chandrasekaran, Sunny C. Jiang

**Affiliations:** Civil and Environmental Engineering, University of California, Irvine, United States of America

**Keywords:** Wastewater reuse, Subsurface irrigation, Virus, Microbial risk assessment, Dynamic model, Transport model

## Abstract

Food production using recycled wastewater offers a sustainable way forward in light of limited freshwater resources. However, concerns of food safety should be addressed to protect public health. To this end, we developed a dynamic transport model to track norovirus from the irrigation water to the root and shoot of lettuce during the growth period. These processes were embodied in a system of ordinary differential equations that also incorporated plant growth, transpiration rate, viral attachment and detachment to culture media, viral decay, and plant barrier effects. Model parameters were either obtained from the literature or through fitting the model to experimental data from a study reporting human norovirus transport in hydroponically grown lettuce. The results showed that lettuce grown hydroponically resulted in a higher risk than lettuce grown in soil. In both cases, the risk predicted failed to meet the risk benchmarks established by the U.S. EPA and WHO. Viral attachment to growth media, such as the soil particles, was an important mechanism for risk reduction. A sensitivity analysis revealed that harvesting time and irrigation time are important factors influencing the viral loads in lettuce. Hence, this pathogen transport model provides a framework for investigating the effects of time and other factors on disease burdens from water reuse in agriculture, underscoring the utility of a dynamic model. In the absence of a routine monitoring of contaminants in the recycled irrigation water and food crops, a quantitative risk assessment based on objective scientific knowledge is the best approach to guide the policy decisions on water reuse practices.

## Highlights

• A dynamic model of viral transport from irrigation water to lettuce was developed.
• Viral attachment to growth medium largely influences lettuce viral load at harvest.
• Norovirus internalized in lettuce poses risk to consumers.
• Risk in hydroponically grown lettuce is much higher than soil grown lettuce.

## Graphical abstract

**Figure:**
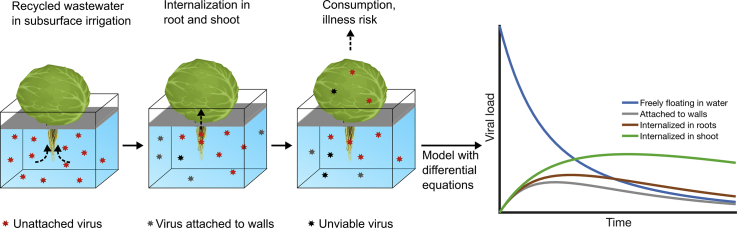

## 1 Introduction

The growth of the human population places an ever-increasing demand on freshwater resources and food supply. The nexus of water and food is now well recognized. One promising strategy to sustain food production in the face of competing water demands is to increase the reuse of treated human wastewater. Municipal wastewater reuse for food production has been successfully adopted in some regions of the world. For example, Israel uses ~84% treated wastewater in agriculture production (Rejwan, 2011). However, Southern California, a region that suffers from a similar degree of water shortage, currently uses less than ~3% of municipal wastewater in agriculture, while discharging ~1.5 million acre-feet effluent per year into the Pacific Ocean (Hauser et al., 2005; Hauser and Hawkins, 2015). Secondary municipal wastewater effluent for ocean discharge is often sufficient to support both the nutrient and water needs for food production. Water reuse in agriculture can bring municipal water reclamation effluent to nearby farms within the city limit, thus promoting local agriculture and also reducing the rate of farmland loss to urban development.

While the use of reclaimed water in agriculture offers a multitude of societal and agronomical benefits, broader adoption faces great challenges. One of the important challenges is ensuring the safety of food products in light of a plethora of human pathogens that may be present in recycled wastewater. Past studies have identified risks associated with irrigating food with recycled wastewater through the retention of the irrigation water on edible plant surfaces during overhead irrigation (Barker et al., 2013; Hamilton et al., 2006; Lim and Jiang, 2013; Mara and Sleigh, 2010; Mok et al., 2014; Sales-Ortells et al., 2015). With the emphasis on water conservation and reduction of evapotranspiration, subsurface drip irrigation is gaining popularity (Tindula et al., 2013). Since there is lesser contact between water and the plant surface, the chance of surface contamination of pathogens is reduced. However, this new practice presents risk of uptake of microbial pathogens into plants. Such internalized pathogens are of greater concerns as washing, even with disinfectants, may not affect pathogens sheltered in the vasculature. Although pathogen transport through root uptake and subsequent internalization into the plant has been a growing research area, results vary due to differences in experimental design, systems tested, and pathogens and crops examined (Carducci et al., 2015; Deering et al., 2012; DiCaprio et al., 2012; Hirneisen et al., 2012; Islam et al., 2004; Rababah and Ashbolt, 2000; Urbanucci et al., 2009; Wei et al., 2011; Wright et al., 2013).

Among the array of pathogens causing foodborne illness that may be carried by treated wastewater, viruses are of the greatest concern but least studied. According to the CDC, 60% of U.S. foodborne outbreaks associated with eating leafy greens were caused by noroviruses (NoV), while *Salmonella* and *E. coli* only accounted for 10% of the outbreaks (http://www.cdc.gov/features/norovirus/). Estimates of global foodborne illness prevalence associated with NoV (~124 million) surpass all other pathogens considered (Havelaar et al., 2015). Viruses are also of concern because they persist in secondary wastewater effluents in high concentrations (da Silva et al., 2007; Flannery et al., 2012). They do not settle well in sedimentation basins and are also more resistant to degradation than bacteria (Wigginton et al., 2012). Therefore, in the absence of solid scientific understanding of the risks involved, the public are likely less receptive to adopting treated wastewater for agricultural irrigation.

NoV internalization in hydroponic systems has been quantified by DiCaprio et al. (2012). Internalization in crops grown in soil is considered lesser (Wei et al., 2011) but nevertheless occurs. However, the only risk assessment (Sales-Ortells et al., 2015) that considered the possibility of NoV internalization in plants assumed a simple ratio of viruses in the feed water over viruses in produce at harvest to account for internalization. The time dependence of viral loads in lettuce was not explored and such an approach did not permit insights into the key factors influencing viral uptake in plants.

In this study, we introduce a viral transport model to predict the viral load in crisphead lettuce at harvest given the viral load in the feed water. It is parameterized for both hydroponic and soil systems. We demonstrate its utility by performing a quantitative microbial risk assessment (QMRA). Strategies to reduce risk enabled by such a model are explored, and a sensitivity analysis highlights possible factors affecting risk.

## 2 Materials and methods

### 2.1 Model structure

**Fig. 1:**
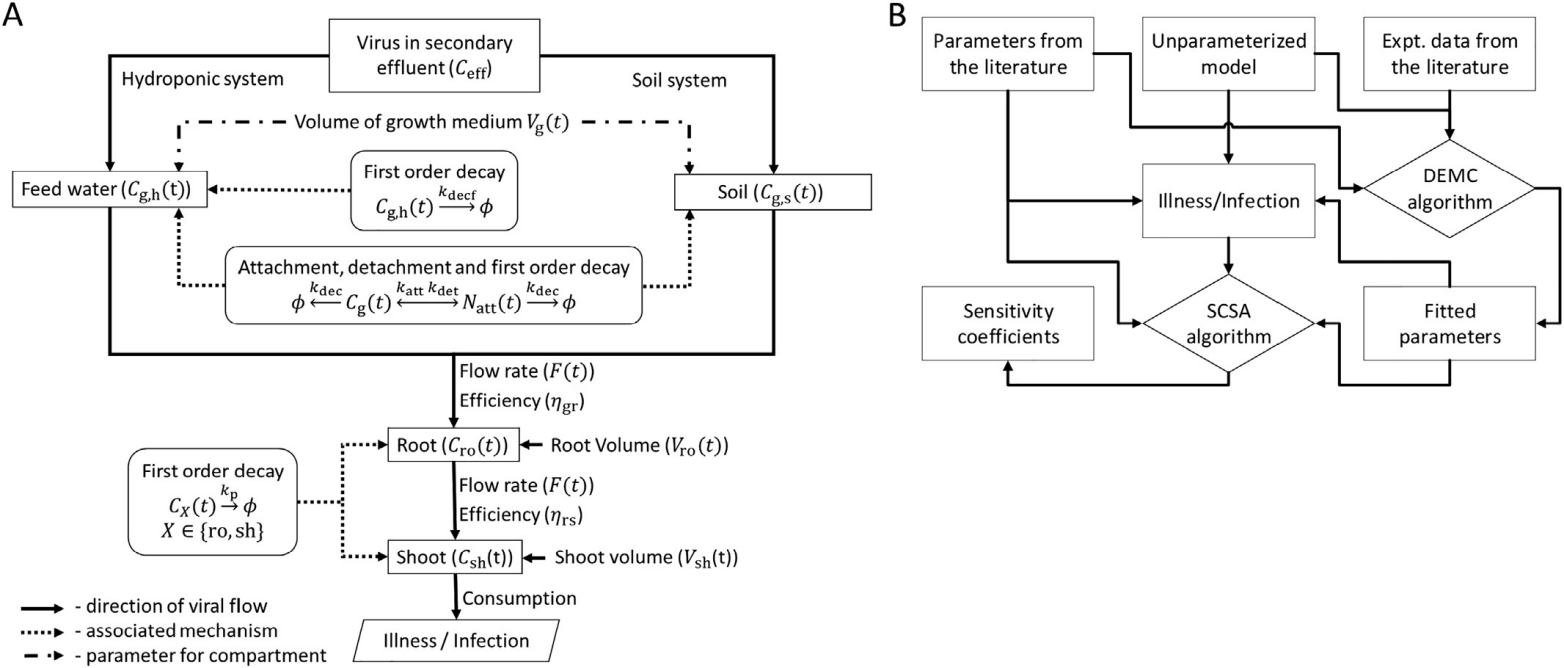
Overview of unparameterized model components (panel A) and statistical techniques applied to the model (panel B). Here *ϕ* represents the null species. Symbols are defined in Table 2.

To understand viral transport from treated human sewage to lettuce through internalization, and the final viral concentration in the plant tissue at the time of consumption, a conceptual transport model is developed (Fig. 1, symbols in Table 2).

In this system, we assumed that the treated wastewater used for cultivating lettuce is secondary sewage effluent that contains *C*_eff_ of NoV/ml. Viral concentrations in the growth medium (*C*_g, h_(*t*) and *C*_g, s_(t) for hydroponic and soil, respectively) at any given time are related to the volume of the growth medium (*V*_g_(*t*)) and the viral removal. Viral removal in the hydroponic growth medium may be modeled simply as a first order decay (Eq. 1) or also to include attachment-detachment (AD) of viruses onto the walls of the hydroponic tank (tank effects, Eq. 2–3). Similarly, we considered AD to soil particles as an important process determining the fraction of viruses transferred from the soil to the plant roots. To avoid making assumptions on the tank geometry, the attached viral load was expressed in viral numbers (*N*_att_(*t*)), which does not depend on the volume or surface area. The viral concentrations in the root (*C*_ro_(*t*)) and shoot (*C*_sh_(*t*)) depend on: 1) the viral transport rate (*F*(*t*)) from the growth medium to the plant, 2) the volumes of these compartments (*V*_ro_(*t*) and *V*_sh_(*t*)), which change with time as the plant grows, and 3) the natural decay of viruses in plant tissues. In addition, two viral transfer efficiencies (*η*_gr_, between growth medium and root and *η*_rs,_ between root and shoot) were included to simulate the potential “barrier” of viral transfer between each compartment (Fig. 1).

Consequently, the viral transport from the growth medium to the root and shoot through internalization was modeled as mass transport through three (growth medium, root and shoot) homogenously well mixed reactors (*n* − 1, *n* and *n* + 1) in series with changing volume. Thus, the generic equation governing viral concentration (*C*_*n*_(*t*)) in the reactor *n* at time *t* is:

$$V_{n}\left(t\right)\frac{dC_{n}\left(t\right)}{\mathrm{dt}}+C_{n}\left(t\right)\frac{dV_{n}\left(t\right)}{\mathrm{dt}}=\eta_{n-1,n}F\left(t\right)C_{n-1}\left(t\right)-\eta_{n,n+1}F\left(t\right)C_{n}\left(t\right)-\text{Removal}\left(C_{n}\left(t\right)\right)$$

where *η*_*n*, *n*+1_ is the efficiency of viral transfer between reactors *n* and *n* + 1. The full model equations specific to individual model components are given by Eq. 1–9 in Table 1 and used for both soil and hydroponic systems. The health risk was finally estimated from consumption of shoot of lettuce irrigated by recycled wastewater (Fig. 1).

**Table 1:** Equations used in the study (all symbols are defined in Table 2).

#	Description	Equation	References
**Rate of change in viral conc** (count cm^−3^ day^−1^)			
1^h^	Growth medium (first order decay)	$\frac{dC_{g}\left(t\right)}{\mathrm{dt}}=-\frac{\eta_{\mathrm{gr}}F\left(t\right)C_{g}\left(t\right)}{V_{g}\left(t\right)}-k_{\text{decf}}C_{g}\left(t\right)-\frac{C_{g}\left(t\right)}{V_{g}\left(t\right)}\frac{dV_{g}\left(t\right)}{\mathrm{dt}}$	This paper
2	Growth medium (first order decay with att-det)	$\frac{dC_{g}\left(t\right)}{\mathrm{dt}}=-\frac{\eta_{\mathrm{gr}}F\left(t\right)C_{g}\left(t\right)}{V_{g}\left(t\right)}-\frac{C_{g}\left(t\right)}{V_{g}\left(t\right)}\frac{dV_{g}\left(t\right)}{\mathrm{dt}}-\left(k_{\mathrm{att}}+k_{\mathrm{dec}}\right)C_{g}\left(t\right)+\frac{k_{\det}N_{\mathrm{att}}\left(t\right)}{V_{g}\left(t\right)}$	
3^a^	Surface attached	$\frac{dN_{\mathrm{att}}\left(t\right)}{\mathrm{dt}}=k_{\mathrm{att}}C_{g}\left(t\right)V_{g}\left(t\right)-\left(k_{\det}+k_{\mathrm{dec}}\right)N_{\mathrm{att}}\left(t\right)$	
4	Root	$\frac{dC_{\mathrm{ro}}\left(t\right)}{\mathrm{dt}}=\frac{\eta_{\mathrm{gr}}F\left(t\right)C_{g}\left(t\right)}{V_{\mathrm{ro}}\left(t\right)}-\frac{\eta_{\mathrm{rs}}F\left(t\right)C_{\mathrm{ro}}\left(t\right)}{V_{\mathrm{ro}}\left(t\right)}-k_{p}C_{\mathrm{ro}}\left(t\right)-\frac{C_{\mathrm{ro}}\left(t\right)}{V_{\mathrm{ro}}\left(t\right)}\frac{dV_{\mathrm{ro}}\left(t\right)}{\mathrm{dt}}$	
5	Shoot	$\frac{dC_{\mathrm{sh}}\left(t\right)}{\mathrm{dt}}=\frac{\eta_{\mathrm{rs}}F\left(t\right)C_{\mathrm{ro}}\left(t\right)}{V_{\mathrm{sh}}\left(t\right)}-k_{p}C_{\mathrm{sh}}\left(t\right)-\frac{C_{\mathrm{sh}}\left(t\right)}{V_{\mathrm{sh}}\left(t\right)}\frac{dV_{\mathrm{sh}}\left(t\right)}{\mathrm{dt}}$	
6^h^	Hydroponic viral transport rate (cm^3^day^−1^)	$F\left(t\right)=a_{t}+b_{t}\left(\rho_{\text{shoot}}d_{\text{shoot},h}\frac{dV_{\mathrm{sh}}\left(t\right)}{\mathrm{dt}}\right)$	(Ciolkosz et al., 1998)

**Growth rates** (cm^3^ day^−1^)			
7^h^	Root, hydroponic	$\frac{dV_{\mathrm{ro}}\left(t\right)}{\mathrm{dt}}=\frac{\exp\left(r_{1}+r_{2}t+r_{3}t^{2}\right)}{\rho_{\text{root},h}d_{\text{root},h}}$	(Both, 1995)
8^h^	Shoot, hydroponic	$\frac{dV_{\mathrm{sh}}\left(t\right)}{\mathrm{dt}}=\frac{\exp\left(s_{1}+s_{2}t+s_{3}t^{2}\right)}{\rho_{\text{shoot}}d_{\text{shoot},h}}$	
9^s^	Shoot, soil	$\frac{dV_{\mathrm{sh}}\left(t\right)}{\mathrm{dt}}=r_{g}V_{\mathrm{sh}}\left(t\right)\left(1-\frac{V_{\mathrm{sh}}\left(t\right)\rho_{\text{shoot}}}{w_{f}}\right)$	(Tei et al., 1996)

**Others**			
10	Daily consumption dose	*λ*_*k*_ = *B* × *L* × *C*_sh_(*t*_ht_) × *ρ*_shoot_^−1^	This paper
11	Approximate beta Poisson	$P_{\inf,k}=1-{\left(1+\frac{\lambda_{k}}{\beta_{B}}\right)}^{-\alpha_{B}}$	(Van Abel et al., 2016)
12	1F1 hypergeometric	*P*_inf, *k*_ = 1−_1_*F*_1_(*α*_H_, *α*_H_ + *β*_H_, −*λ*_*k*_)	
13	Fractional Poisson	$P_{\inf,k}=P_{F}\left(1-\exp\left(-\frac{\lambda_{k}}{\mu_{a}}\right)\right)$	
14	Annual infect. risk	$P_{\inf,\mathrm{ann}}=1-\prod_{k=1}^{365}\left(1-P_{\inf,k}\right)$	(Karavarsamis and Hamilton, 2010)
15	Annual illness risk	*P*_ill, ann_ = *P*_ill∣inf_*P*_inf, ann_	(Mara and Sleigh, 2010)
16	Annual disease burden	*D*_annual_ = *P*_ill∣inf_*P*_ill, ann_*D*_p_	
17^s^	Volume of growth medium (soil)	*V*_g, s_ = *V*_e_*θ*	(Clapp and Hornberger, 1978)

a Units of (count day^−1^).

h Specific to hydroponic model.

s Specific to soil model.

### 2.2 Model parameters to estimate viral transport in lettuce

Some parameters to complete the conceptual viral transport model were obtained from the literature. Others were estimated by fitting the model to published data from experiments using NoV seeded feed water to grow crisphead lettuces in a hydroponic system (DiCaprio et al., 2012). The initial volume of 800 mL for the hydroponic growth medium (*V*_g, h_(0)) was adopted based on these experiments. The volume reduction over time was assumed equivalent to the plant transpiration rate (Ciolkosz et al., 1998) between refills. For the soil system, the volume of the growth medium (*V*_*g*, *s*_) equals the volume of water contained in the soil interstitial spaces in an envelope around the roots. This envelope is a region (of volume *V*_e_) around the roots that the plant is assumed to interact with. *V*_*g*, *s*_ is given by Eq. 17, where *θ* is the volumetric water content obtained from Clapp and Hornberger (1978). Estimates for *V*_e_ spanned a large range (Supplementary S1E) and a middle value of *V*_e_ = 80000 cm^3^ was adopted and assumed to be constant over the lettuce growth period. This assumed value was also verified to have minimal impact on the model outcome (see 2.5, 3.2).

The plant transpiration rate was adopted as the viral transport rate (*F*(*t*)) based on: 1) previous reports of passive bacterial transport in plants (Bell et al., 1995; Warriner et al., 2003; Wilson, 1995), 2) the significantly smaller size of viruses compared to bacteria, and 3) the lack of known specific interactions between human viruses and plant hosts (Supplementary S1A). Accordingly, viral transport rate in hydroponically grown lettuce (Eq. 6) was determined from the previously reported transpiration model (Ciolkosz et al., 1998), in which the transpiration rate is proportional to the lettuce growth rate and is influenced by cultivar specific factors (*a*_t_, *b*_t_). These cultivar specific factors used in our model were predicted using the hydroponic crisphead lettuce growth experiment carried out by DiCaprio et al. (2012) described in Section 2.3 (and Supplementary S1D). Since the transpiration rate in soil grown lettuce is significantly higher than that in the hydroponic system, viral transport rate in soil grown lettuce was obtained directly from the graphs published by Gallardo et al. (1996) using WebPlotDigitizer (Rohatgi, 2015) (Supplementary S1G for details).

The growth rates of lettuce root $\left(\frac{dV_{\mathrm{ro}}\left(t\right)}{\mathrm{dt}}\right)\;$and shoot $\left(\frac{dV_{\mathrm{sh}}\left(t\right)}{\mathrm{dt}}\right)\;$in hydroponic systems were estimated using Eq. 7–8 (parameters in Table 2; details and assumptions in Supplementary section S1B, S1C). The shoot growth rate (in terms of fresh volume) for soil grown lettuce was determined using Eq. 9 (parameters in Table 2; details in Supplementary section S1F). In the absence of a published root growth model for lettuce in soil, a fixed root volume of 100 cm^3^ was used.

**Table 2:** Summary of symbols and parameter values.

Parameter	Symbol	Value/distribution (units)	References
**Common (soil + hydroponic)**			
Body weight^b^	*B*	[67.0 (10.7, 113.9)] (kg person^−1^)	(Kahn and Stralka, 2009)
Viral load in effluent^b^	*C*_eff_	[4.13 (0.04, 624.32)] (count mL^−1^)	Lim et al. (2016)
Concentration of virus in growth medium (root, shoot)^a^	*C*_g_(*t*) (*C*_ro_(*t*), *C*_sh_(*t*))	*C*_g, s_(0) = *C*_g, h_(0) = *C*_eff_ (count mL^−1^)	
DALYs per case of NoV GE	*D*_p_	9 × 10^−4^ (person^−1^year^−1^)	(Kemmeren et al., 2006)
Volumetric flow rate^a^	*F*(*t*)	*F*_h_(*t*) from Eq. (6); *F*_s_(*t*):from plot (mL day^−1^)	(Both, 2003; Gallardo et al., 1996)
Growth medium viral attachment rate^a^	*k*_att_	*k*_att, s_(S1, S2) = 4.1, 0.8; *k*_att, h_ [f] (day^−1^)	(Schijven et al., 1999)
Growth medium viral decay rate (with attachment-detachment)^a^	*k*_dec_	*k*_dec, s_ = 0.15 *k*_dec, h_ [f] (day^−1^)	(Roberts et al., 2016)
Growth medium viral decay rate (only first order decay)	*k*_decf_	[f] (day^−1^)	
Growth medium viral detachment rate^a^	*k*_det_	*k*_det, s_(S1, S2) = 8.7 × 10^−4^, 3.0 × 10^−3^ *k*_det, h_[f] (day^−1^)	(Schijven et al., 1999)
Viral decay constant in plant (root, shoot)	*k*_p_	[f] (day^−1^)	
Consumption of lettuce per kg bodyweight^b^	*L*	[0.38, (0.04, 2.08)] (g lettuce kg^−1^person^−1^day^−1^)	(U.S. EPA, 2003)
Number of viruses attached to growth medium	*N*_att_(*t*)	Model intermediate (count)	
Probability of illness if infected	*P*_ill∣inf_	0.8	(Moe, 2009)
Last irrigation time^a^	*t*_li_	*t*_li, h_ = 21; *t*_li, s_ = 56 (days)	(Both, 2003; Gallardo et al., 1996)
Harvest time^a^	*t*_ht_	*t*_ht, h_ = 35; *t*_ht, s_ = 70 (days)	
Volume of growth medium (root, shoot)^a^	*V*_g_(*t*) (*V*_ro_(*t*), *V*_sh_(*t*))	*V*_g, h_(0)=800; *V*_ro, s_=100 (mL)	
Fractional Poisson risk model parameters	*P*_F_, *μ*_a_	0.72, 1	(Van Abel et al., 2016)
Beta Poisson risk model parameters	*α*_B_, *β*_B_	0.104, 32.3	
Hypergeometric risk model parameters	*α*_H_, *β*_H_	0.46, 1.20	
Growth medium - root transfer efficiency	*η*_gr_	[f]	
Root - shoot transfer efficiency	*η*_rs_	[f]	
Shoot density	*ρ*_shoot_	0.35 (g cm^−3^)	(Jenni and Bourgeois, 2008)

**Hydroponic specific**			
Rate parameters for viral transport by plant	*a*_t_, *b*_t_	[f] (mL day^−1^, mL)	
Ratio of dry to fresh weight of lettuce root	*d*_root, h_	0.057	Estimated from (Zhang et al., 2015)
Ratio of dry to fresh weight of lettuce shoot	*d*_shoot, h_	0.045	(Both, 2003; Ciolkosz et al., 1998)
Root growth constants	*r*_1_	−8.482	
	*r*_2_	0.4586 (day^−1^)	
	*r*_3_	−6.472 × 10^−3^(day^−2^)	
Shoot growth constants	*s*_1_	−7.414	
	*s*_2_	0.406 (day^−1^)	
	*s*_3_	−5.579 × 10^−3^(day^−2^)	
Root density	*ρ*_root, h_	0.2 (g cm^−3^)	Assumed

**Soil specific**			
Shoot growth constant	*r*_g_	0.2056 (day^−1^)	(Tei et al., 1996)
Envelope volume	*V*_e_	80000 (cm^3^)	Assumed
Final weight of lettuce	*w*_f_	550 (gm)	(Jenni and Bourgeois, 2008)
Volumetric water content of soil	*θ*	0.435	(Clapp and Hornberger, 1978)

[f]: Fitted values, presented in Table 3.

a These are represented by their subscripts (h: hydroponic, s: soil) where required.

b Empirical distributions, values presented are median (95% interval).

In the viral transport model, viral transfer efficiency (*η*) was used to account for the potential “barrier” between each compartment (i.e. root and shoot). The existence of such a “barrier” is evident from field experiments where some microbial pathogens were internalized in the root but not in the shoot of plants (Oron et al., 1995). In addition, viral transfer efficiencies (ranging from 0 to 1) also account for differing observations in pathogen internalization due to the type of pathogen or lettuce. For example, DiCaprio et al. (2012) reported the internalization of NoV into lettuce, while Urbanucci et al. (2009) did not detect any NoV in another type of lettuce grown in feed water seeded with viruses. The values of *η*_gr_ and *η*_rs_ were determined by fitting the model to experimental data reported by DiCaprio et al. (2012) and is detailed in Section 2.3. The values of *η*_gr_ and *η*_rs_ predicted for the hydroponic lettuce were assumed in the soil case.

The viral removal in the growth medium includes both die-off and AD, while only natural die-off was considered in the lettuce root and shoot. AD kinetic constants (*k*_att, h_, *k*_det, h_) as well as the growth medium viral decay constant (*k*_dec, h_) in the hydroponic case were obtained by fitting the model to the data from DiCaprio et al. (2012). Viral AD in soil has been investigated in both lab scale soil columns and field studies (Schijven and Hassanizadeh, 2000). In our model, viral AD constants (*k*_att, s_, *k*_det, s_) in soil were obtained from the experiments of Schijven et al. (1999), who investigated MS2 phage kinetics in sandy soil in field experiments. As the MS2 phage was transported with the water in soil, the AD rates changed with the distance from the source of viruses. To capture the range of AD rates, two scenarios of viral behavior in soils were investigated. Scenario 1 (Sc1) used the AD rates estimated at the site closest to the viral source (well 1), while scenario 2 (Sc2) used data from the farthest site (well 6). In contrast to lab scale soil column studies, field studies provided more realistic viral removal rates (Schijven and Hassanizadeh, 2000). Using surrogate MS2 phage for NoV provided conservative risk estimates since MS2 attached to a lesser extent than NoV in several soil types (Meschke and Sobsey, 1998). The viral decay rate in the soil (*k*_dec, s_) determined by Roberts et al. (2016) was adopted because the experimental temperature (20–30 °C) and soil type (clay loam) are more relevant to lettuce growing conditions compared to the other decay study (Schijven et al., 1999). Decay rates in the root and shoot were used from the hydroponic system predictions.

### 2.3 Model fitting for viral transport in hydroponic grown lettuce

The transport model was fitted to log_10_ viral concentration data from DiCaprio et al. (2012), extracted from graphs therein using WebPlotDigitizer (Rohatgi, 2015). In these experiments, NoV of a known concentration was spiked in the feed water (growth medium) of hydroponic lettuce and was monitored in the feed water, the root and shoot over time. While fitting the model, an initial feed volume of 800 mL (as used in the experiments) was adopted and parameters producing final volumes of <200 mL were rejected.

To fit the model while accounting for uncertainty in the data, a Bayesian approach was used to maximize the likelihood of the data given the parameters. A posterior distribution of the parameters was obtained by the differential evolution Markov chain (DE-MC) (Ter Braak, 2006) algorithm, which can be parallelized and can handle multimodality of the posteriors distribution without fine tuning the jumping distribution. Computation was carried out on MATLAB R2016a (Mathworks) and its ParCompTool running on the High Performance Computing facility at UC Irvine. The code is available at https://github.com/JiangLabUCI/ViralTransport.

Table 3 lists the parameters estimated by model fitting and their search bounds. Fitting data from DiCaprio et al. (2012) without including viral AD to the tank walls was attempted but the results were not used in the risk estimates due to the poor fit of model to the data. The rationale behind the model fitting procedure and diagnostics are discussed in Supplementary section S1H.

**Table 3:** Parameter values predicted from fitting model to data from hydroponic experiments.

Parameter	Units	Search bounds	Median (95% credible interval)
*a*_t_	cm ^3^ day^−1^	(0,100)	19.82 (0.71,39.92)
*b*_t_	cm^3^ g^−1^	(0,300)	40.10 (1.19,96.96)
*η*_gr_	–	(0,1)	0.48 (0.07,0.97)
*η*_rs_	–	(0,1)	0.74 (0.24,0.99)
*k*_att, h_	day^−1^	(0,20)	10.66 (0.62,19.55)
*k*_det, h_	day^−1^	(0,10)	5.19 (0.65,9.76)
*k*_dec, h_	day^−1^	(0,100)	0.25 (0.03,0.54)
*k*_p_	day^−1^	(0,20)	0.54 (0.02,6.29)

### 2.4 Risk estimation for consumption of lettuce

The initial viral concentration (*C*_eff_) in the irrigation water was drawn from an empirical distribution reported previously by Lim et al. (2016) for NoV in activated sludge treated secondary effluent. As justified by Lim et al. (2016), the sum of the concentrations of two genotypes known to cause illness was used to construct the distribution. The NoV concentrations in lettuce shoot were estimated at typical harvest times: *t*_ht, h_ = 35 days in the hydroponic system and *t*_ht, s_ = 70 days in soil. We also assumed the last irrigation with recycled water occurred on *t*_li, h_ = 21 days for hydroponic and *t*_li, s_ = 56 days for soil grown lettuce. Together with parameters in Table 2, Table 3, these values were used in Eq. 1–9 (Table 1) to generate the probability distribution of NoV concentration for hydroponic or soil grown lettuce on MATLAB R2016a (Mathworks).

To estimate the risk from consumption of lettuce, the daily viral dose was computed using Eq. 10 (Table 1) for the *k*^th^ day. The body weight (*B*) was drawn from an empirical distribution for all ages and genders in the United States, from a report of the percentile data of body weight. The lettuce consumption rate (*L*) was drawn from an empirical distribution constructed from data reported by the Continuing Survey of Food Intakes by Individuals (CSFII). The ‘consumer only’ data for all ages and genders was used, and hence the reported risk is only for those who consume lettuce. It is important to note that the daily viral dose was computed in (count g^−1^) from the model output (in count mL^−1^) using the shoot density *ρ*_shoot_ (Eq. 10) to be consistent with the consumption rate reported in CSFII.

Several different NoV dose-response models have been proposed based on limited clinical data. The validity of these models is a matter of much debate (Schmidt, 2015; Van Abel et al., 2016), which is beyond the scope of this study. These models differ in their assumptions resulting in large variability of predicted risk outcomes. To cover the range of potential outcomes of human exposure to NoV, we estimated and compared risk outcomes using three models: 1) Approximate Beta-Poisson (BP) (Teunis et al., 2008; Van Abel et al., 2016); 2) Hypergeometric (_1_*F*_1_) (Mcbride, 2014; Atmar et al., 2014; Teunis et al., 2008); and 3) Fractional Poisson (FP) (Messner et al., 2014). In the risk estimation, we considered NoV in the lettuce tissue exists as individual viral particles (disaggregated form) and used the disaggregated NoV models. The model equations are given by Eq. 11–13, Table 1. Ten thousand samples of the daily infectious risks were calculated from BP and FP models using MATLAB R2016a. Wolfram Mathematica 11.1 (Wolfram Research) was used for the (_1_*F*_1_) model estimation as it was faster.

Using a random set of 365 daily risk estimates of (*P*_inf, *k*_ for day *k*), the annual infection risk (*P*_inf, ann_) was calculated according to the Gold Standard estimator (Karavarsamis and Hamilton, 2010) using Eq. 14, Table 1. While there appears to be some dose dependence for illness resulting from infection *P*_ill∣inf_ (Atmar et al., 2014; Teunis et al., 2008), this has not been clearly elucidated for the different dose response models. Hence, we adopted the procedure used in Mara and Sleigh (2010) and calculated annual illness risk with Eq. 15. The annual disease burden in terms of DALY (disability adjusted life years) lost per case (*D*_p_) was set to 9 × 10^−4^ pppy for each case of NoV disease (Kemmeren et al., 2006). The annual disease burden (*D*_annual_) is given by Eq. 16 in Table 1. As part of the risk characterization process, we compared risk outcomes of this study to risk benchmarks established by the U.S. EPA and WHO for acceptable level of health risk and the estimates by Sales-Ortells et al. (2015).

### 2.5 Sensitivity analysis

Global sensitivity of the log_10_(*P*_inf_) (daily risk) to input parameters was determined by the SCSA method (Li et al., 2010) since it accounts for correlation in input parameters (not handled by Sobol's method (Sobol, 2001)). Three sensitivity indices were produced for each parameter, the structural (S_struct_), correlative (S_corr_) and total (S_tot_) sensitivities. Fitted parameters were used as is, maintaining the observed correlation structures. Parameters drawn from distributions were varied within their 95% credible intervals while other parameters spanned ranges obtained from literature (Supplementary S1I). The MATLAB implementation of SCSA by Sahin and Vrugt (personal communication) was used.

## 3 Results

### 3.1 Model fitting and parameter prediction

**Fig. 2:**
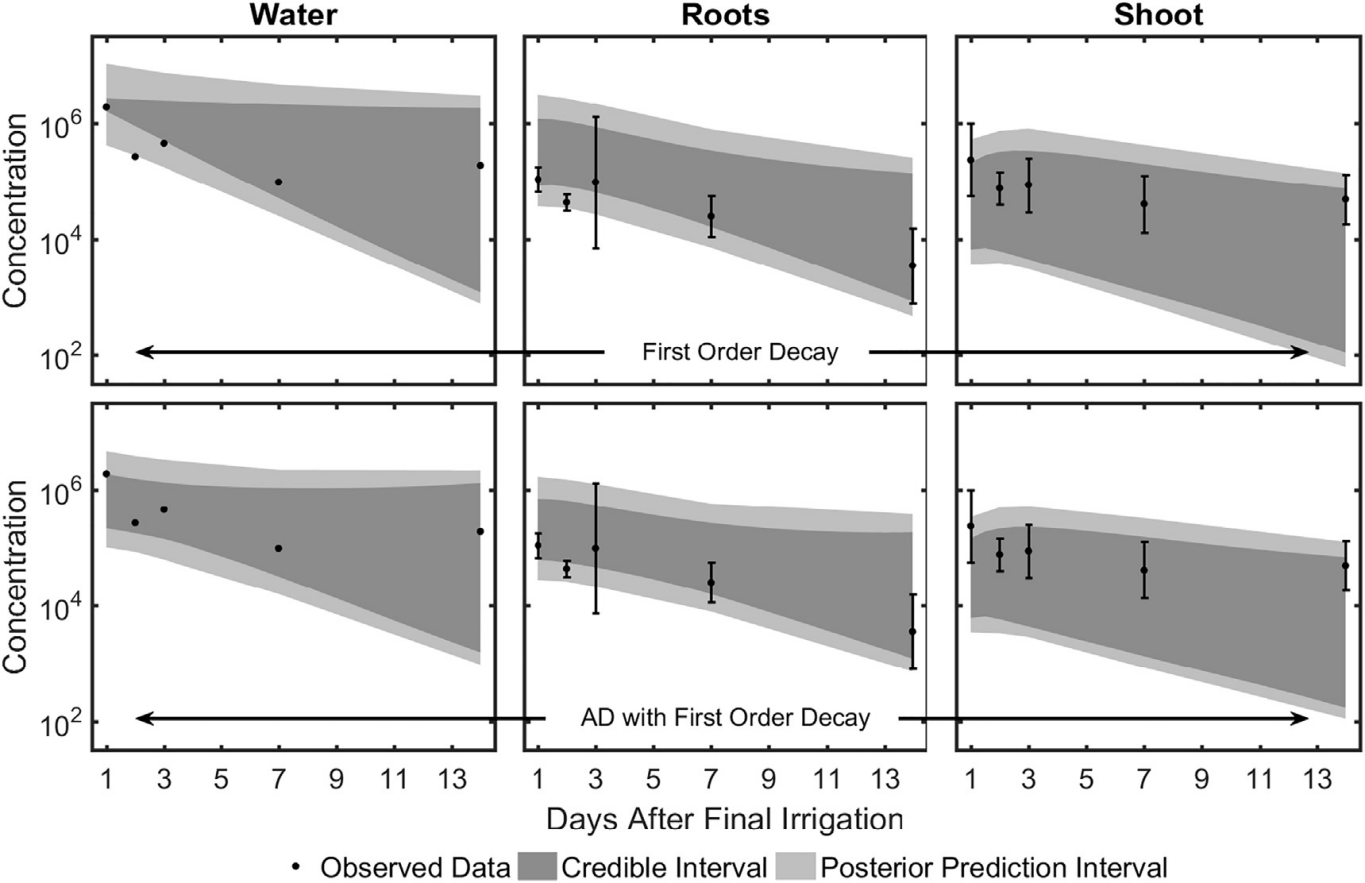
Fit of the model to data from DiCaprio et al. (2012) for lettuce grown in hydroponic system. Top panel shows model fitting using first order viral decay only; bottom panel shows model fitting using first order viral decay and viral attachment/detachment (AD) to cultivation tank wall in growth medium (water). Error bars indicate standard deviations of 3 samples.

A summary of the model fitting exercise for viral transport in hydroponic grown lettuce is presented in Fig. 2. Under the assumption of first order viral decay, NoV loads in water (growth medium) at two time-points did not fall in the credible region of model predictions, indicating that mere first order decay was unsuitable to capture the observed viral concentration data. The addition of the AD factor into the model addressed this inadequacy and importantly supported the curvature observed in the experimental data. This result indicates the AD of viruses to hydroponic tank wall is an important factor to include in predicting viral concentration in all three compartments (water, root, shoot). Credible and prediction intervals in the shoot at harvest were similar for both models.

**Fig. 3:**
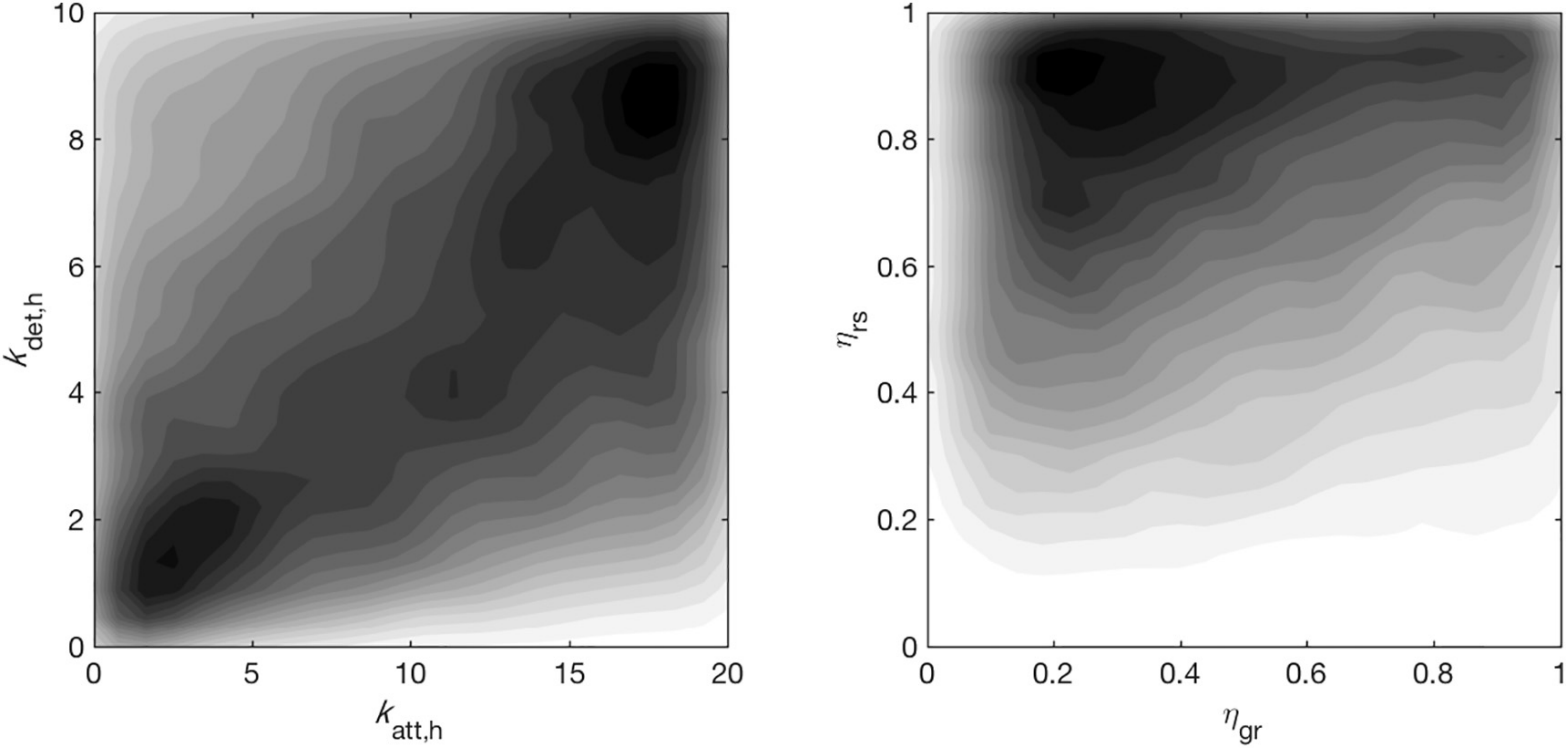
Illustration of joint distributions of posterior samples from fitting the model with AD of viruses to hydroponic tank walls. The shaded gradients (light to dark) indicate the localization of parameters in sub-regions of the initial search space, illustrating coupling between the parameters.

The adequacy of model fit was also revealed by the credible intervals of the predicted parameters for the model with AD (Table 3). Four of the predicted parameters: *a*_t_, *b*_t_, *k*_dec, s_ and *k*_p_, were restricted to a smaller subset of the search bounds, indicating that they were identifiable. In contrast, the viral transfer efficiency *η* and the kinetic parameters (*k*_att, h_, *k*_det, h_) spanned the entirety of their search space and were poorly identifiable. However, this does not suggest that each parameter can independently take any value in its range because the joint distributions of the parameters (Fig. 3) indicate how fixing one parameter (e.g. *η*_gr_ = 0.9) influences the likelihood of another parameter (*η*_rs_ most likely to be closer to 1). Hence, despite the large range of an individual parameter, the coordination between the parameters constrained the model predictions to produce reliable outcomes (Section 4.1, Supplementary Fig. S6). Therefore, the performance of the model with AD was considered adequate for estimating parameters used for risk prediction.

### 3.2 Health risks from lettuce consumption

**Fig. 4:**
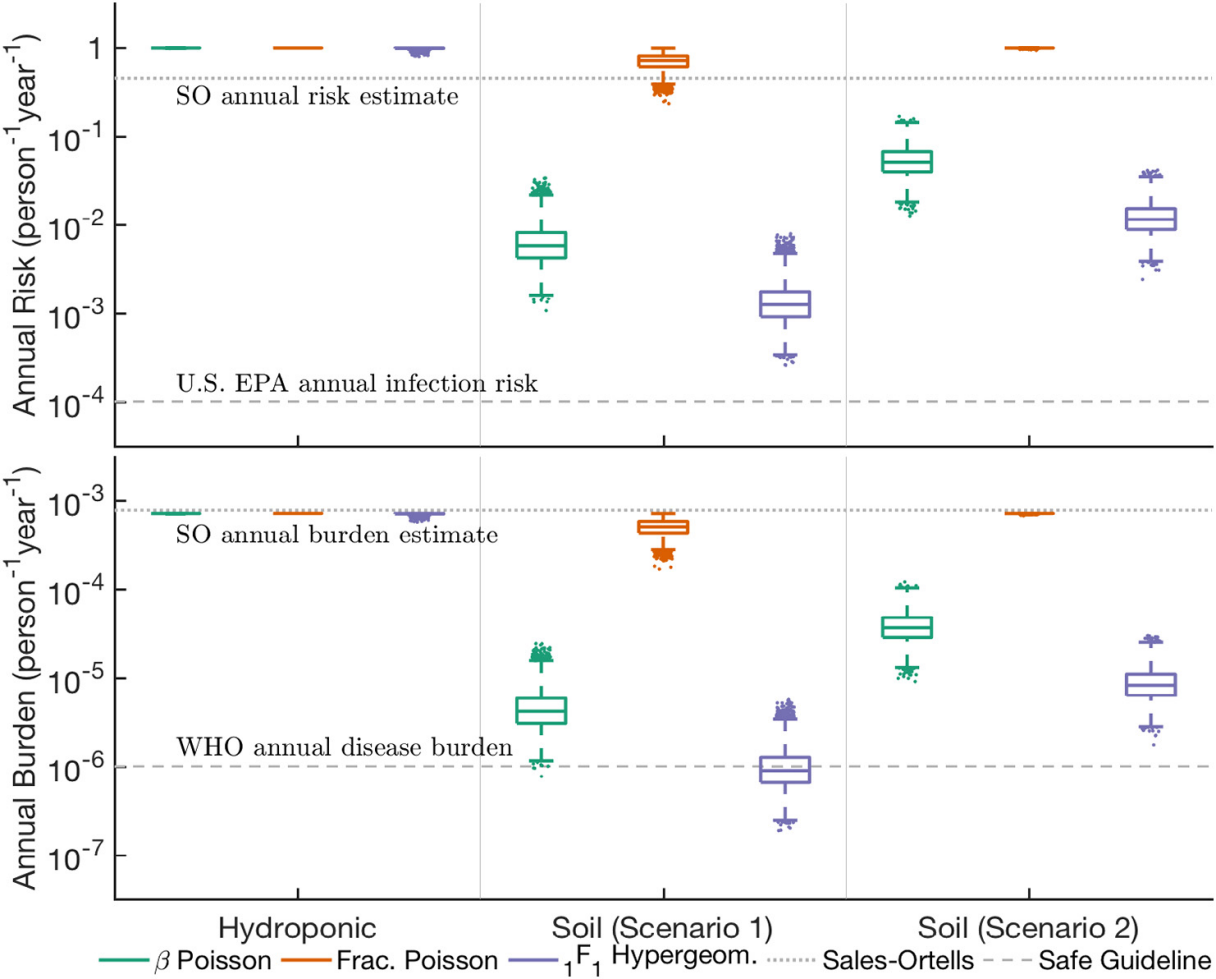
Annual risk (top panel) and disease burden (bottom panel) of norovirus infection through consumption of lettuce grown hydroponically or in soil (scenarios 1 and 2 explained in Section 2.2) using treated sewage effluent. The dashed lines indicate existing risk benchmarks or the mean plotted from a previous study by Sales-Ortells et al. (2015).

Risk estimates for lettuce grown in the hydroponic tank or soil are presented in Fig. 4. Across these systems, the FP model predicted the highest risk while the 1F1 model predicted the lowest risk. For a given risk model, higher risk was predicted in the hydroponic system than in the soil. This is a consequence of the very low detachment rates in soil compared to the attachment rates. Comparison of results from Sc1 and Sc2 (session 2.2) of soil grown lettuce indicated lower risks and disease burdens under Sc1 (Fig. 4).

Comparing with the safety guidelines, the lowest risk predicted in the hydroponic system is higher than the U.S. EPA defined acceptable annual drinking water risk of 10^−4^ (Environmental Protection Agency, 2010) for each risk model. The annual burdens are also above the 10^−6^ benchmark recommended by the WHO (2011). In the case of soil grown lettuce, neither Sc1 nor Sc2 met the U.S. EPA safety benchmark. Two risk models predicted borderline disease burden according to the WHO benchmark, for soil grown lettuce in Sc1, but under Sc2 the risk still did not meet the safety guideline. Neither increasing holding time of the lettuce to two days after harvesting nor using bigger tanks significantly altered the predicted risk (Supplementary Fig. S2). In comparison, the risk estimates of Sales-Ortells et al. (2015) are higher than range of soil grown lettuce outcomes presented here (Fig. 4) for 2 of 3 models.

**Fig. 5:**
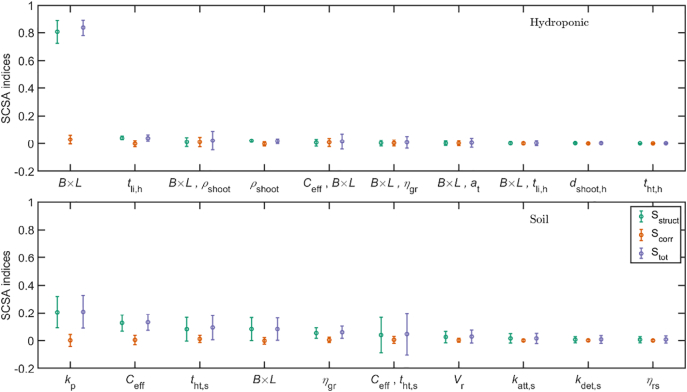
Top 10 most significant SCSA sensitivity indices (mean ± s.d., 100 bootstrapped samples) for hydroponic (top panel) and soil (bottom panel) grown lettuce. *S*_struct_, *S*_corr_ and *S*_tot_ correspond to S_a_, S_b_ and S in (Li et al., 2010). A comma denotes second order sensitivity terms for pairs of parameters.

The SCSA sensitivity indices are presented in Fig. 5. For hydroponically grown lettuce, the top 3 factors (by S_tot_) influencing daily risk are amount of lettuce consumed, time since last irrigation and the term involving consumption and *ρ*_shoot_. Also, the risk estimates are robust to the fitted parameters (Table 3) despite low identifiability of some model parameters (*a*_*t*_, *b*_*t*_, *k*_att, h_ and *k*_det, h_ ). For soil grown lettuce, *k*_p_ appears to be the major influential parameter, followed by the input viral concentration in irrigation water and the lettuce harvest time. S_corr_ is near zero, suggesting lesser influence of correlation in the input parameters.

## 4 Discussion

In this study, we modeled the internalization and transport of NoV from irrigation water to the lettuce using ordinary differential equations to capture the dynamic processes of viral transport in lettuce. This first attempt is aimed at underscoring the importance of the effect of time in determining the final risk outcome. The modeling approach from this study may be customized for other scenarios for the management of water reuse practices and for developing new guidelines for food safety. Moreover, this study identifies critical gaps in the current knowledge of pathogen transport in plants and calls for further lab and field studies to better understand risk of water reuse.

### 4.1 Fitting model to data

To develop an adequate model to predict viral transport in plant tissue, it is necessary to couple mathematical assumptions with an understanding of the underlying biogeochemical processes governing virus removal, plant growth, growth conditions and virus-plant interactions. For example, although a simple transport model without AD could predict the viral load in the lettuce at harvest, it failed to capture the initial curvature in the viral load in the growth medium (water). An alternative to the AD hypothesis that could capture this curvature is the existence of two populations of viruses as used in Petterson et al. (2001), one decaying slower than the other. However, a closer examination of the double exponential model revealed that it was not time invariant. This means that the time taken to decay from a concentration *C*_1_ to *C*_2_ is not unique and depends on the history of the events that occurred (Supplementary Fig. S3). Other viral models, such as the ones used in Peleg and Penchina (2000) faced the same issues. The incorporation of AD made the model time invariant and always provided the same time for decay between two given concentrations. This model fitting experience showcases how mathematics can guide the understanding of biological mechanisms. The hypothesis of two different NoV populations is less plausible than that of viral attachment and detachment to the hydroponic tank. While it appears that incorporating the AD mechanism does not significantly improve viral load prediction in lettuce shoot at harvest, this is a consequence of force fitting the model to data under the given conditions. Changing the conditions, for example, by reducing viral attachment rate to the tank wall, will underestimate virus load in the lettuce shoot in the absence of AD (Supplementary Fig. S4). Through this model fitting exercise, we also acknowledge that the model can be significantly improved with new insights on virus-plant interactions and more data on the viral transport through plants.

A potential cause for concern in the model fit is the wide intervals. However, there is significant uncertainty in the data as well (e.g. root day 3, shoot day 1, Fig. 2) suggesting that the transport process itself is noise prone. Moreover, from the perspective of risk assessment, the variability between dose-response models is higher than the within dose-response model variability (Supplementary Fig. S6). Since within dose-response model variability stems from uncertainty in viral loads at harvest among other factors, the wide intervals do not exert a bigger effect than the discordance from different dose response models.

### 4.2 Model parameter estimates

Some parameters (i.e., *k*_dec, h_, *k*_p_) are identifiable to a good degree through model fitting, but there is a large degree of uncertainty in the viral transport efficiencies and the AD kinetic parameters. While this could be a consequence of fitting limited number of data points with several parameters, the viral load at harvest and risk estimates were well constrained. This large variation in parameters and ‘usefully tight quantitative predictions’ (Supplementary Fig. S6) is termed the sloppiness of parameter sensitivities, and has been observed in physics and systems biology (Gutenkunst et al., 2007; Waterfall et al., 2006). Well-designed experiments may simultaneously reduce uncertainty in the parameters as well as predictions (Apgar et al., 2010; Chachra et al., 2011), and therefore increasing confidence in predictions. One possible experiment to reduce parameter uncertainty is recording the transpiration and growth rate to fit Eq. 6 independently to acquire *a*_t_ and *b*_t_.

### 4.3 Risk estimates

An interesting outcome of our analysis is the strong association of risk with plant growth conditions. The health risks from consuming lettuce irrigated with recycled wastewater are highest in hydroponic grown lettuce, followed by soil grown lettuce under Sc2 and the least in soil grown lettuce under Sc1 (Fig. 4 and Section 2.2). This difference in risk estimates stems to a large degree from the difference in AD kinetic constants (*k*_att, s_, *k*_det, s_) (Supplementary Fig. S5). Increasing *k*_att, s_ (holding *k*_det, s_ constant) will decrease risk as more viruses will get attached to the growth medium, while increasing *k*_det, s_ (holding *k*_att, s_ constant) will have the opposite effect (Supplementary Fig. S5), as more detached viruses are available for uptake by the plant. The combined effect of the AD parameters depends on their magnitudes and is portrayed in Supplementary Fig. S5. This result indicates that a better understanding of the virus interaction with the growth environment can lead to an improved understanding of risk. More importantly, this outcome indicates that soil plays an important role in the removal of viruses from irrigation water through adsorption of viral particles. An investigation focused on understanding the influence of soil composition on viral attachment will help refine the transport model.

The risk predicted by this dynamic transport model is greater than the EPA annual infection risk as well as the WHO annual disease burden benchmark. The reasons for this outcome are many-fold. First, there is a significant variability in the reported internalization of viruses in plants. In research of data for modeling NoV transport in plant, we filtered the existing data using the following criteria: 1) human NoV used as the seed agent, 2) quantitative viral results in growth medium and different locations of the plant. Based on these criteria, the data from DiCaprio et al. (2012) represent the best available data on viral internalization and transport in lettuce. However, it is also important to note that a similar study by Urbanucci et al. (2009) did not observe human NoV internalization in lettuce. This discrepancy could be due to the specific subspecies of the plant and growth conditions used in the studies. In addition, minor changes such as damages in roots or decrease in humidity of the growing environment can promote pathogen internalization (Hirneisen et al., 2012; Ward and Mahler, 1982). Alternatively, tracking viral transport through the growth medium and plant is challenging, which may yield false results due to reaction inhibitions in genome amplification and poor detection limit.

The risk outcome of this study is conservative because it assumes an individual consumes the wastewater irrigated lettuce daily for an entire year. This assumption and the corresponding higher risk estimates are only applicable to a small portion of consumers, while most consumers in the U.S. are likely to have a more diverse diet. While the model outcomes presented here represent the best attempt given the available data, it is also possible that the internalization observed by DiCaprio et al. (2012) is an extreme case and typical internalization is lesser.

As previously discussed by others (Schmidt, 2015; Van Abel et al., 2016), risk estimates by different NoV dose-response models differed by orders of magnitude. This study primarily aims to introduce a viral transport model without advocating any one dose-response model. We hope the future refinement of pathogen dose-response models will reduce variability in risk estimates.

The risk of consuming lettuce grown in soil as predicted by Sales-Ortells et al. (2015) is higher than our predictions, although the results of DiCaprio et al. (2012) were used in both studies. This is a consequence of considering the greater adsorption capability of soil, which is not reflected when assuming a simple input:output ratio. Using different inoculating concentrations of NoV, body weight and consumption rate distributions also contributed to difference in the outcomes but to a lesser extent.

Parameters for crisphead lettuce were obtained from several different sources, each possibly using a different sub-variety of cripshead. Yet, global sensitivity analysis showed insensitivity of risk estimates to several assumed and fitted parameters (*a*_t_, *b*_t_, *d*_root, h_, *V*_e_), lending confidence to the approaches taken to parametrize the model. The importance of taking the dynamics of viral transport is underscored by the sensitivity to *t*_li, h_ in hydroponic and *t*_ht, s_ in soil cases. This suggests that given no change in lettuce consumption, changes in irrigation schedule can affect risk outcome. Such arguments were not possible with the approach of Sales-Ortells et al. (2015). In soil grown lettuce, the high sensitivity to *k*_p_ indicate the role of plant specific processes in mediating risk outcome.

### 4.4 Contribution and future directions

In addition to a transport model predicting the NoV load in lettuce, we explore the strategies to reduce the risk of NoV gastroenteritis (Supplementary Fig. S2) by increasing holding time of the produce after harvesting or using bigger hydroponic culture volumes. Although neither strategy could significantly alleviate the risks, the process highlights two strengths of modeling: 1) It provides mathematical support for arguments that would otherwise be less convincing; 2) It predicts outcomes of experiments without the physical resources required to perform them. For instance, the model can be used to explore alternate irrigation schedules to reduce the NoV internalization risk.

Modeling also helps encapsulate our understanding of the system and generate hypotheses. For example, simple first order decay did not produce the trend observed in the water, which suggests that additional mechanisms are at play. We postulate the attachment of virus particles on the walls of the hydroponic system as one possible mechanism and examined the fit of the model. Although viral attachment to glass or other materials has been observed before (Boche and Quilligan Jr., 1966), here it stands as a hypothesis that can be tested. In addition to generating and testing hypotheses, some of our model assumptions raise broader questions for future research. For example, it was assumed that viruses are transported at the rate of transpiration from the growth medium to the roots, yet not much is known regarding the role of roots in the internalization of viruses. Investigating the defense mechanisms of plants' roots to passive viral transport, i.e. through rhizosphere microbiome interactions, may shed light on the broad understanding of plant and microbe interactions.

The question of extending this model to other pathogen and plant systems draws attention to the dearth of data in enabling such efforts. While modeling another virus may not require changes to the model, understanding transport in other plants can be challenging. Data required includes models for growth rate and transpiration, plant growth characteristic including density, water content, as well as internalization studies to determine transport efficiencies. However, from the perspective of risk management, lettuce may be used as the worst-case scenario estimate of risk in water reuse owing to its high consumption with minimal pathogen inactivation by cooking. This worst-case scenario can be used to set water quality standards for irrigation water for production of fresh produce eaten raw. The models can also be extended to include pathogen transport to the plant tissue from manure/biosolids that are used as organic fertilizer.

## 5 Conclusion

A dynamic viral transport model was developed to predict the viral load in the lettuce at harvest, given the viral load in the recycled water used for irrigation. Integrating the viral load with the exposure model and NoV dose-response models, we estimated the annual infection risk and disease burden through consumption of lettuce irrigated with recycled wastewater. The following conclusions are made from the study:

1. Viral transport in the plant depends on its interaction with the growth medium and the plant tissue to a large extent.
2. The experimental data reported in literature are best explained by the incorporation of attachment and detachment of the viruses to the cultivation tank.
3. Kinetic rates for attachment and detachment as well as transport efficiencies between plant compartments were loosely constrained in their search bounds (low identifiability). However, both joint distributions of the parameters and the final risk predictions were well constrained, highlighting the sloppy parameter sensitivities.
4. The overall risk estimates from the model are greater than the commonly acceptable infection risk benchmark or annual disease burden. However, there are large uncertainties in the experimental data of viral transport through plants.
5. The model provides a foundation to incorporate new data on pathogen transport and plant-microbe interactions to develop a holistic understanding of pathogen internalization.

## Conflicts of interest

Authors declare no conflict of interest.

## Appendix A Supplementary data

Supplementary material Image 1
