# Supplementary material for: A dynamic transport model for quantification of norovirus internalization in lettuce from irrigation water and associated health risk
Source: Sci Total Environ. 2018 Dec 1;643:751–61. doi: 10.1016/j.scitotenv.2018.06.158 (PMC6138827; doi:10.1016/j.scitotenv.2018.06.158)
Supplement: Supplementary file 1 — Supplementary material [file mmc1.docx]

**Supplementary Information:**

**A dynamic transport model for quantification of norovirus internalization in lettuce from irrigation water and associated health risk**

Srikiran Chandrasekaran^1^ and Sunny Jiang^1^*

^1^Civil and Environmental Engineering, University of California, Irvine

*Corresponding author: 844E, Engineering Tower, University of California, Irvine, CA 92697; Tel: 949-824-5527; email: [sjiang@uci.edu](mailto:sjiang@uci.edu)

***Section S1***

**Hydroponic growth system**

1. **Justifying the setting of rate of volume reduction to rate of transpiration**

Water is lost from the hydroponic tank by two processes, evaporation and transpiration. Ciolkosz et al. (1998) used generalized linear models to fit the rate of evapotranspiration (ET) in hydroponically grown lettuce. They found that the air temperature and vapor pressure deficit had the lowest significance among the covariates considered. Taken together with the marked increase in water usage as the lettuce grows, the evaporation component in ET is thought to be less important in the hydroponic system. Hence, we made a simplifying assumption that the rate of ET, rather than the rate of transpiration, drives viral transport. We equated the flow rate to the rate of ET for these reasons.

1. **Volume of shoot**

The growth model for the lettuce shoot dry weight (given below) was obtained from Ciolkosz et al. (1998), which was first introduced in Both (1995). The dry weight was converted to fresh weight using the dry:fresh weight ratio $d_{shoot,h}$=0.045 (Both, 2003) and shoot density $\rho_{\mathrm{shoot}}$=0.35 g/cm^3^ (Jenni and Bourgeois, 2008).

$$M_{\mathrm{shoot}}^{'}(t)=exp(s_{1}+s_{2}t+s_{3}t^{2})$$

$M_{\mathrm{shoot}}^{'}(t)$ is rate of accumulation of dry mass (g dry wt/ mL). $s_{1}$, $s_{2}$ and $s_{3}$ are as defined in Table 2. Volumetric growth rate is given by Eqn 8, Table 1.

1. **Volume of roots**

The growth model for the lettuce root dry weight was obtained from Both (2003). This was converted to fresh weight using the dry:fresh weight ratio ($d_{root,h}$=0.057) estimated from Zhang et al. (2015a) white LED with no supplemental light). A density $\rho_{root,h}$=0.2 g/cm^3^ was assumed.

$$M_{\mathrm{root}}^{'}(t)=exp(r_{1}+r_{2}t+r_{3}t^{2})$$

$M_{\mathrm{root}}^{'}(t)$ is rate of accumulation of dry mass (g dry wt/ mL). $r_{1}$, $r_{2}$ and $r_{3}$ are as defined in Table 2. Volumetric growth rate is given by Eqn 7, Table 1.

1. **Water volume fitting considerations**

While Ciolkosz et al. (1998) found that the rate of dry mass accumulation had the highest significance, we could not use the coefficients ($a_{t}, b_{t}$) they provided. This is because the cumulative water usage predicted by those coefficients in the monitored period is higher than the tank volume of 800 mL specified by DiCaprio et al. (2012). To account for the lower water usage, we fitted the coefficients while maintaining the conclusion of the experiment that the rate of ET is a linear function of the dry mass accumulation rate.

**Soil growth system**

1. **Volume of envelope**

The reported soil envelope volume ($V_{e}$) in the lettuce Rainha De Maio in Portas (1973) for different soil types varied from 3332 – 8568 $cm^{3}$ although the growth stage is not reported. In Weaver and Bruner (1927), a different cultivar (Early Prize Head) was used and very different estimates are obtained, especially for the later stages of growth. Fitting a cone of radius 3.46 inches and height 6 inches in the early stage gives a volume of 3706$cm^{3}$. Fitting a cone of radius 1.5 feet and height 5 feet gives a volume of 3.336$\times{10}^{5} cm^{3}$ for the mature stage. Using these numbers as guidelines, we fixed the soil envelope volume ($V_{e}$) to 80000 $cm^{3}$ and verified the low sensitivity of the risk estimates to large variations in this parameter (Fig 5).

1. **Volume of shoot**

Tei et al. (1996a) compared 3 lettuce growth models – the logistic, Gompertz (Gompertz, 1825) and the expolinear (Goudriaan & Monteith 1990) model. We chose the logistic model (Eq 8, Table 1), despite its higher root mean squared error, because there are insufficient data in simulating the expolinear and Gompertz models.

This model predicts dry weight/area but can be extended to fresh volume per unit plant because: 1) The number of plants per unit area (n_p_=17.6 plants/m^2^) remains constant over the course of that experiment (Tei et al., 1996b); 2) The ratio of dry weight: fresh weight ($d_{shoot,s}$) remains constant over the lettuce growth phase (Both, 2003); 3) The density of the shoot ($\rho_{\mathrm{shoot}}$) remains constant over the growth phase. The growth equation in dry weight/area ($W$) over time is given by:

$$\frac{dW(t)}{dt}=r_{g}W(t)(1-\frac{W(t)}{W_{f}})$$

where $W_{f}$ is the final dry weight per unit area. Substituting $W(t)=V_{\mathrm{sh}}(t)n_{p}d_{shoot,s}\rho_{\mathrm{shoot}}$ and $W_{f}=w_{f}n_{p}d_{shoot,s}$ (where $w_{f}$ is the final fresh weight per plant), we get Eqn 8, Table 1. Hence the same growth rate constant $r_{g}$is applicable for the fresh volume model. The final fresh weight ($w_{f}$) and density ($\rho_{\mathrm{shoot}}$) were taken from Jenni and Bourgeois (2008) for the Ithaca variety.

1. **Flow rate**

Gallardo et al. (1996) used a model to predict the transpiration rate (in $mm day^{-1}$) of lettuce over its growth phase in soil. To get the volumetric flow rate, we multiply by an arbitrary area and divide by the number of plants in that area:

$$F \left( mL plant^{-1}\mathrm{da}y^{-1} \right)= ROT\left( mm day^{-1} \right)\times\frac{area}{\# plants in that area}$$

where $F$ is the flow rate and $ROT$ is the rate of transpiration. Since lettuce was reportedly to occupy three fourths of the total area at harvest time (the rest occupied by soil), we compute the per plant transpiration rate by considering an area of 1$m^{2}$ using:

$$F \left( mL plant^{-1}\mathrm{da}y^{-1} \right)= \frac{ROT\left( cm day^{-1} \right)}{10}\times\frac{1m^{2}}{(0.75m^{2}\div area of fully grown lettuce)}$$

The ROT are obtained from figure 3 in Gallardo et al. (1996) using WebPlotDigitizer (Rohatgi, 2015) and converted to flow rate using the method described above.

1. **Model fitting and diagnostics**

Log­_10_ of the viral concentration was fitted because viral concentrations at different time points differed by orders of magnitude. Fitting concentrations resulted in good fit for early timepoints but inaccurate estimates for later timepoints used in estimating the risk. While maximizing the likelihood of the log_10_ concentration, we did not weight by the standard deviations as the these estimates were from small samples numbers (Motulsky and Christopoulos, 2004). Multi objective approaches to simultaneously maximize the individual likelihoods of water, root and shoot concentrations were not pursued due to the similar orders magnitude of the likelihoods. For the same reason, the likelihoods were not weighted differently.

Convergence diagnostics were also investigated. The first half of the 20000 iterations of DE-MC were discarded (burn-in). Outlier chains were identified by their characteristic higher mean objective function value compared to other chains and discarded. The  $\hat{R}$ values Gelman et al. (2013) of the remaining chains was close to 1 (data not shown). Samples from these chains (after burn-in and outlier removal) were randomly subsampled and used for further analysis.

1. **Sensitivity analysis**

| Parameter | Fixed value | Lower bound | Upper bound | Source |
| --- | --- | --- | --- | --- |
| $\rho_{\mathrm{shoot}}$ | 0.35 | 0.27 | 0.35 | Other lettuce densities in (Jenni and Bourgeois, 2008) |
| $d_{root,h}$ | 0.057 | 0.0444 | 0.071 | 95% quantile by assuming normal distributed root weights (Zhang et al., 2015b) |
| $d_{shoot,h}$ | 0.045 | 0.04 | 0.05 | From (Both, 2003) |
| $\rho_{root,h}$ | 0.2 | 0.1 | 1 | Assumed to span one order of magnitude. |
| $t_{ht,h}$ | 14 | 12 | 16 | Assumed two-day difference |
| $V_{g,h}(0)$ | 800 | 600 | 6000 | Assumed from experiment, span one order of magnitude |
| $t_{li,h}$ | 21 | 19 | 23 | Assumed two-day difference |
| $V_{e}$ | 80000 | 8000 | 80000 | Assumed to span one order of magnitude |
| $\theta$ | 0.435 | 0.435 | 0.476 | Ranging from sandy loam to clay loam (no loamy sand or sand) from Clapp and Hornberger (1978) |
| $k_{dec,s}$ | 0.15 | 0.15 | 0.199 | Surface applied, PBS, 2 soil types from Roberts.et al 2016 |
| $V_{root,s}(t)$ | 100 | 30 | 300 | Assumed to span one order of magnitude |
| $r_{g}$ | 0.2056 | 0.203 | 0.2082 | From standard error in Tei et. al 1996b |
| $w_{f}$ | 550 | 335 | 550 | From Jenni and Bourgeois, 2008 |
| $k_{att,s}$ | 4.1, 0.8 | 0.8 | 4.1 | (Schijven et al., 1999) |
| $k_{det,s}$ | 0.00087, 0.003 | 0.00087 | 0.003 |  |
| $t_{ht,s}$ | 14 | 12 | 16 | Assumed two-day difference |


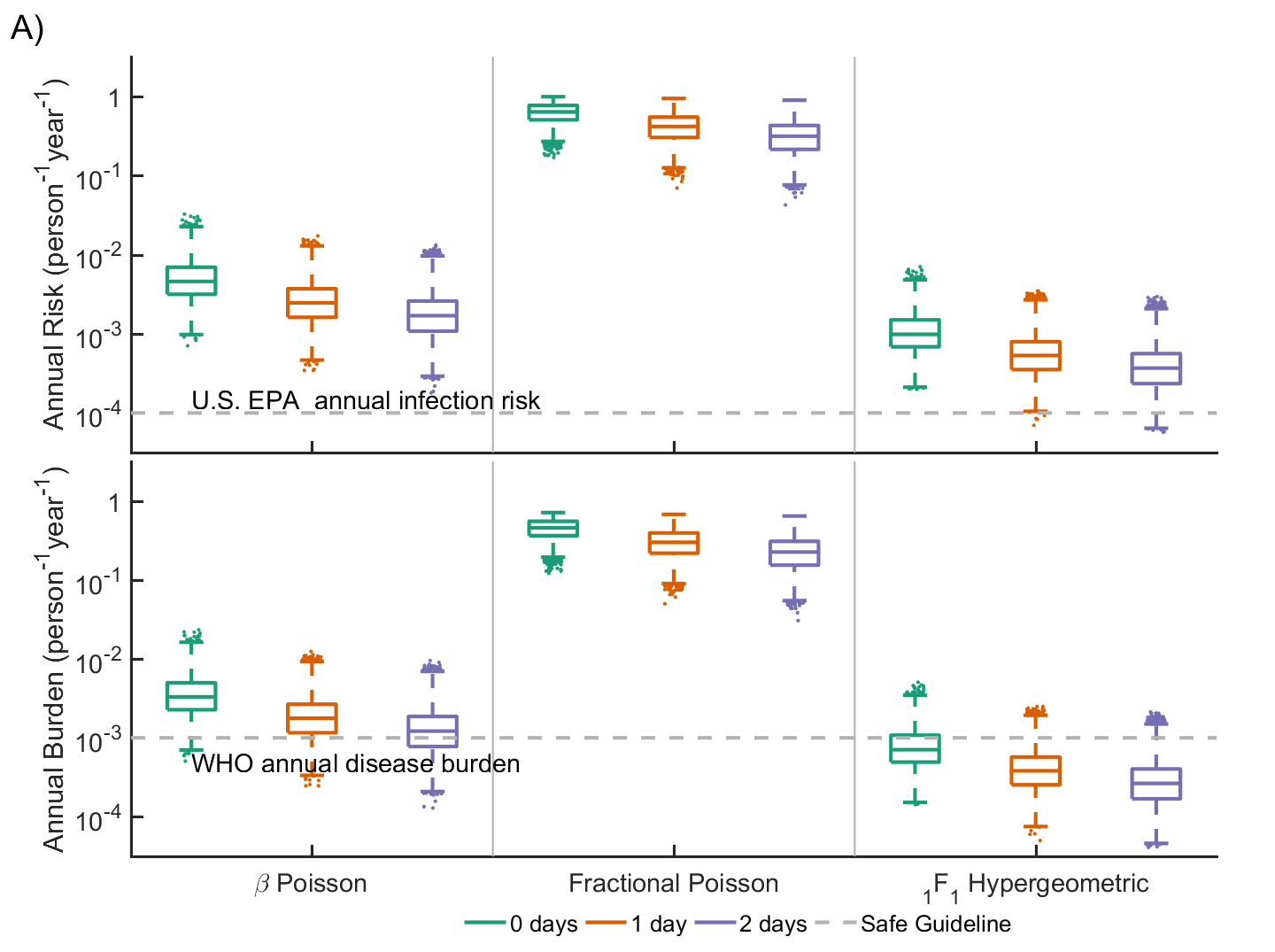

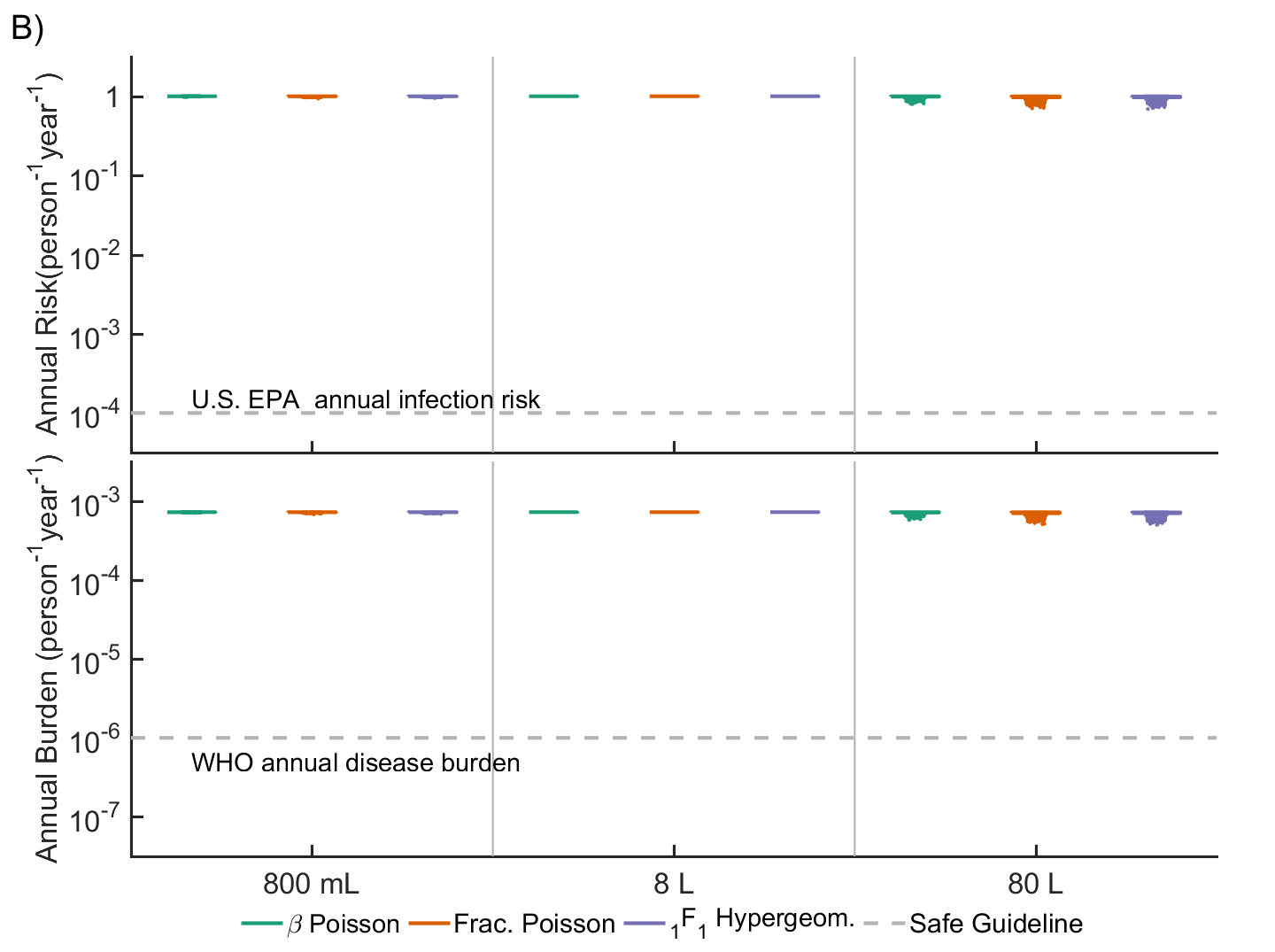


Fig S2. Comparison of risks by increasing holding time of lettuce after harvesting from soil (A) or increasing tank volume of hydroponic grown lettuce (B). Both strategies failed to reduce risk below the acceptable limits.


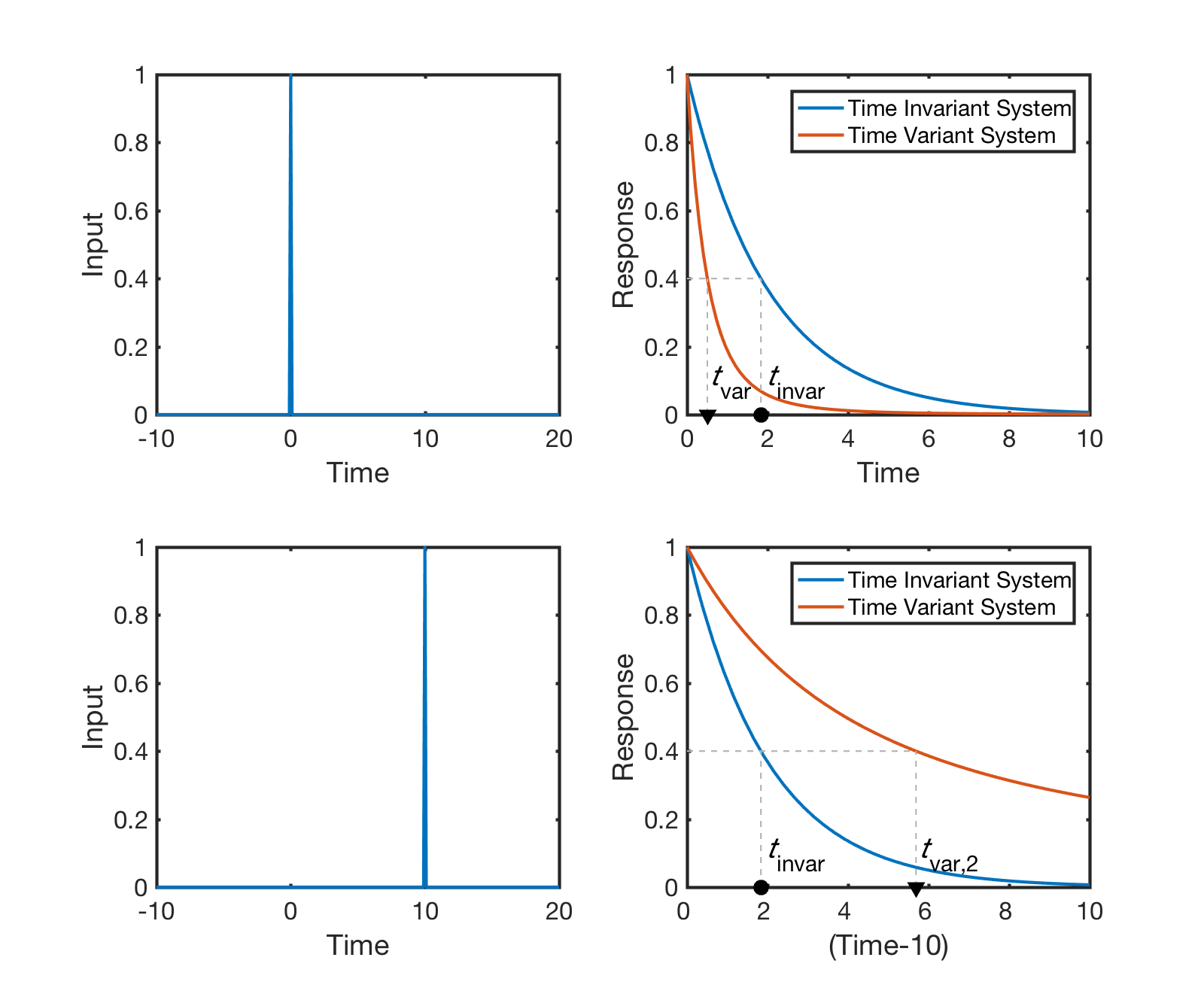


Fig S3. Illustration of the difference in response to delay input for a time invariant vs. a time variant system. An input pulse at t=0 produces the response in the top right panel for time invariant and time variant systems. Shifting the input pulse to t=10 produces a shifted (but same shape of blue lines) response in the time invariant case but a different characteristic response (different shape of orange lines) in the time variant case. Time to reach a response of 0.4 remains in the same relative position ($t_{\mathrm{invar}}$) for the time invariant system whereas it shifts ($t_{\mathrm{var}}$ to $t_{var,2}$) for the time variant system, showing non-unique times for reaching the same response.


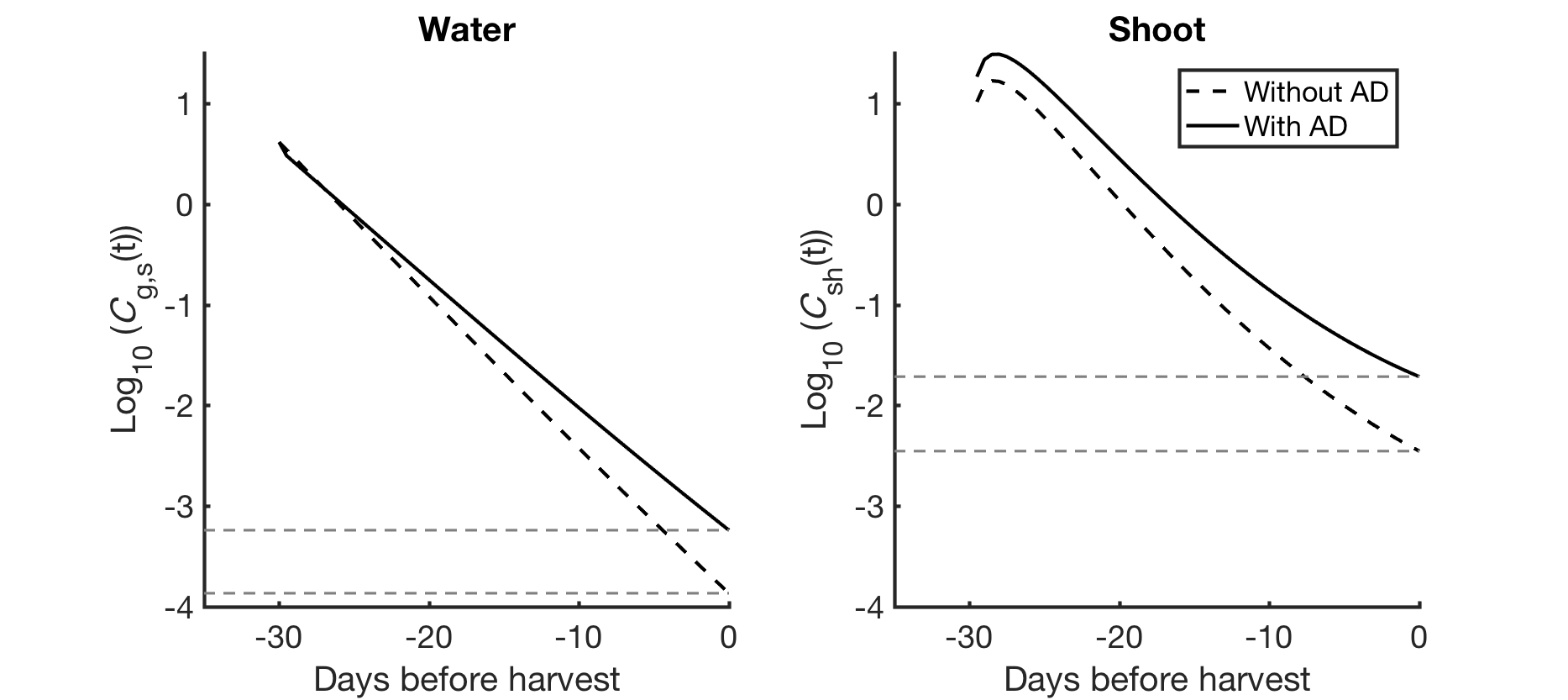


Fig S4. Comparison of the viral concentration in the growth medium (water) and lettuce shoot using models with and without incorporation of AD of viruses to hydroponic tank walls. In this simulation, a lower $k_{att,s}$ value (one tenth of the best fit parameter from the) was used. The model without AD underestimates the viral load in the lettuce.


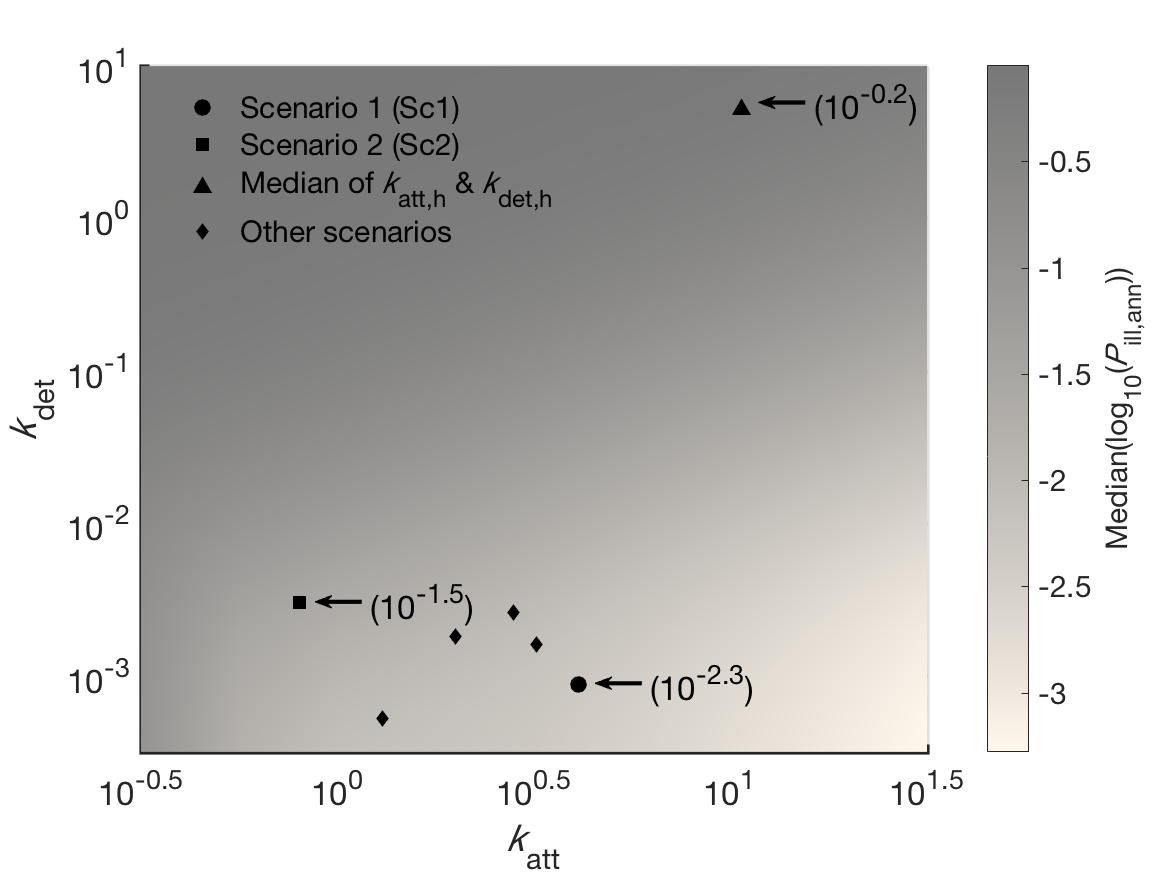


Fig S5. Illustration of median(log_10_(risk)) as a function of AD kinetic parameters for *soil* grown lettuce, $\beta$ Poisson risk model. Simply replacing $k_{att,s}$ and $k_{det,s}$ with the medians of $k_{att,h}$and $k_{det,h}$ in the *soil* model drastically reduces the median(log_10_(risk)). Comparing numerical values of median(log_10_(risk)) listed above in () with Figure 4 (top panel, median of $\beta$ Poisson boxes) shows that a change in AD kinetic parameters exerts a major influence in determining the magnitude of risk.


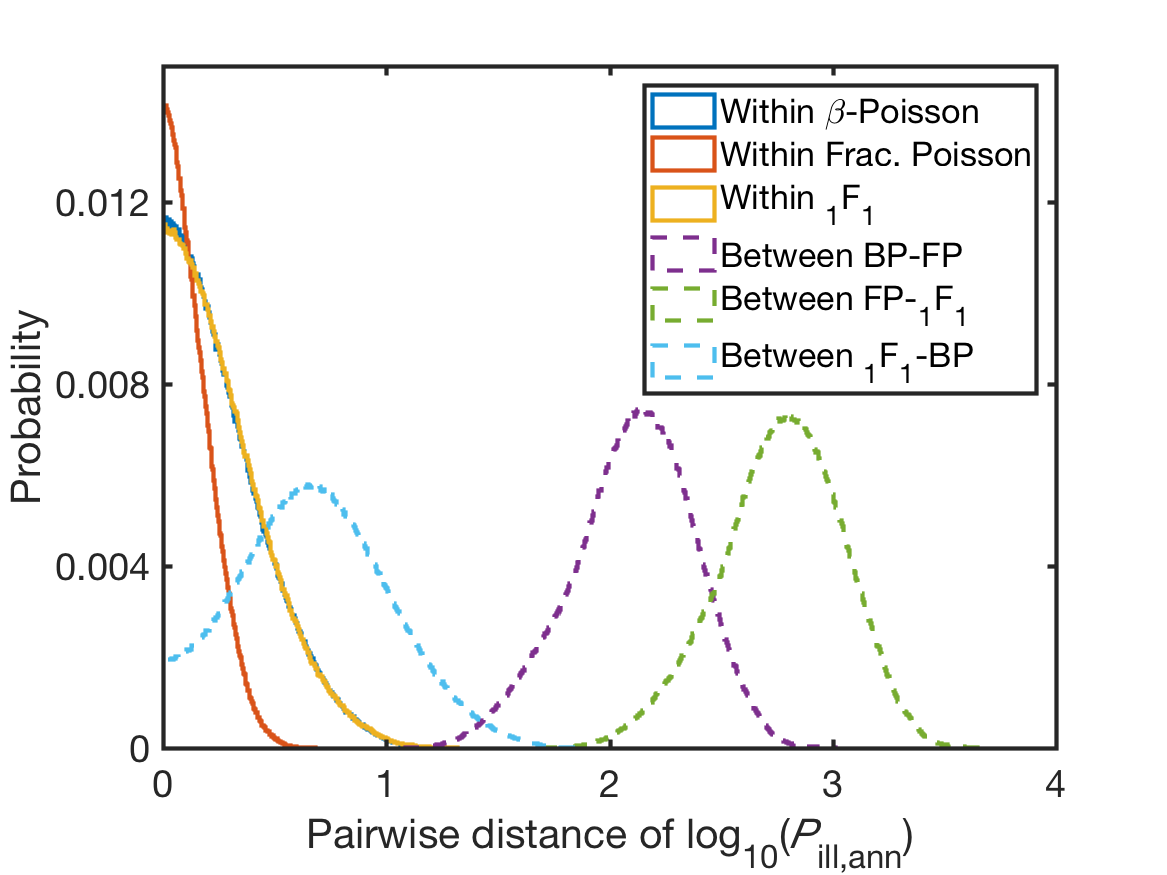


Fig S6. Histograms of the pairwise distances of annual risk estimates (Sc1), $P_{ill,ann}$ for the different dose response models. Pairwise distance is a measure of variability with lower distances indicative of less variability. The within dose-response model variability (solid lines) are lesser than between dose-response model variability (dashed lines).

**Bibliography**

Both, A., 2003. Ten years of hydroponic lettuce research. Knowledgecenter.Illumitex.Com 18, 8.

Both, A.-J., 1995. Dynamic simulation of supplemental lighting for greenhouse hydroponic lettuce production. Cornell University.

Ciolkosz, D.E., Albright, L.D., Both, A.J., 1998. Characterizing evapotranspiration in a greenhouse lettuce crop. Acta Hortic. doi:10.17660/ActaHortic.1998.456.29

Clapp, R.B., Hornberger, G.M., 1978. Empirical equations for some soil hydraulic properties. Water Resour. Res. 14, 601–604. doi:10.1029/WR014i004p00601

DiCaprio, E., Ma, Y., Purgianto, A., Hughes, J., Li, J., 2012. Internalization and dissemination of human norovirus and animal caliciviruses in hydroponically grown romaine lettuce. Appl. Environ. Microbiol. 78, 6143–6152. doi:10.1128/AEM.01081-12

Gallardo, M., Snyder, R.L., Schulbach, K., Jackson, L.E., 1996. Crop Growth and Water Use Model for Lettuce. J. Irrig. Drain. Eng. 122, 354–359. doi:10.1061/(ASCE)0733-9437(1996)122:6(354)

Gelman, A., Carlin, J., Stern, H., Dunson, D., Vehtari, A., 2013. Bayesian data analysis, 3rd ed. Taylor & Francis.

Gompertz, B., 1825. On the Nature of the Function Expressive of the Law of Mortality. Philos. Trans. 27, 513–585.

Goudriaan, J., Monteithf, J.L., 1990. A Mathematical Function for Crop Growth Based on Light Interception and Leaf Area Expansion. Ann. Bot. 66, 695–701.

Jenni, S., Bourgeois, G., 2008. Quantifying phenology and maturity in crisphead lettuce. Horttechnology 18, 553–558.

Motulsky, H., Christopoulos, A., 2004. Fitting models to biological data using linear and nonlinear regression : a practical guide to curve fitting. Oxford University Press.

Portas, C.M., 1973. Development of root systems during the growth of some vegetable crops. Plant Soil 39, 507–518. doi:10.1007/BF00264169

Rohatgi, A., 2015. WebPlotDigitizer.

Schijven, J.F., Hoogenboezem, W., Hassanizadeh, M., Peters, J.H., 1999. Modeling removal of bacteriophages MS2 and PRD1 by dune recharge at Castricum, Netherlands. Water Resour. Res. 35, 1101–1111. doi:10.1029/1998WR900108

Tei, F., Aikman, D.P., Scaife, A., 1996a. Growth of Lettuce, Onion and Red Beet. 2. Growth Modelling. Ann. Bot. 78, 645–652. doi:10.1006/anbo.1996.0172

Tei, F., Scaife, a, Aikman, D.P., 1996b. Growth of Lettuce, Onion, and Red Beet. 1. Growth Analysis, Light Interception, and Radiation Use Efficiency. Ann. Bot. 78, 633–643. doi:10.1006/anbo.1996.0171

Weaver, J.E., Bruner, W.E., 1927. Root development of vegetable crops, Development, McGraw-Hill publications in the agricultural and botanical sciences, E.W. Sinnott, consulting editor. McGraw-Hill Book Company, Incorporated.

Zhang, G., Shen, S., Takagaki, M., Kozai, T., Yamori, W., 2015a. Supplemental Upward Lighting from Underneath to Obtain Higher Marketable Lettuce (Lactuca sativa) Leaf Fresh Weight by Retarding Senescence of Outer Leaves. Front. Plant Sci. 6, 1–11. doi:10.3389/fpls.2015.01110

Zhang, G., Shen, S., Takagaki, M., Kozai, T., Yamori, W., 2015b. Supplemental Upward Lighting from Underneath to Obtain Higher Marketable Lettuce (Lactuca sativa) Leaf Fresh Weight by Retarding Senescence of Outer Leaves. Front. Plant Sci. 6, 1–9. doi:10.3389/fpls.2015.01110
